# The association between dietary inflammation index and depression

**DOI:** 10.3389/fpsyt.2023.1131802

**Published:** 2023-03-23

**Authors:** Ling Luo, Jie Hu, Ruixian Huang, Danli Kong, Wei Hu, Yuanlin Ding, Haibing Yu

**Affiliations:** ^1^The First Dongguan Affiliated Hospital, Guangdong Medical University, Dongguan, Guangdong, China; ^2^Department of Epidemiology and Medical Statistics, School of Public Health, Guangdong Medical University, Dongguan, Guangdong, China; ^3^Department of Maternal, Child and Adolescent Health, School of Public Health, Tongji Medical College, Huazhong University of Science and Technology, Wuhan, Hubei, China; ^4^Department of Epidemiology, School of Public Health, Sun Yat-sen University, Guangzhou, Guangdong, China; ^5^Key Laboratory of Chronic Disease Prevention and Control and Health Statistics, School of Public Health, Guangdong Medical University, Dongguan, Guangdong, China

**Keywords:** dietary inflammatory index, depression, insulin resistance, National Health and Nutrition Examination Survey, mediation analysis

## Abstract

**Objective:**

We aimed to evaluate whether depression is associated with increased risk of dietary inflammatory index (DII) or energy-adjusted DII (E-DII) and whether the association is partly explained by insulin resistance (IR).

**Methods:**

Base on the National Health and Nutrition Examination Survey (NHANES) 2005–2018. Univariate analyses of continuous and categorical variables were performed using *t*-test, ANOVA, and *χ*^2^ test, respectively. Logistic regression was used to analyze the relationship between DII or E-DII and depression in three different models. Mediation analysis was used to assess the potential mediation effects of homeostatic model assessment-IR (HOMA-IR).

**Results:**

A total of 70,190 participants were included, and the DII score was higher in the depressed group. DII score was related to all participant characteristics except age (*p* < 0.05). After being included in covariates (Model 3), participants in the highest quartile of DII score have increased odds of depression (*OR*: 1.82, 95% *CI*: 1.28–2.58) compared with those in the first quartile of DII score. And, a significant dose–response relationship was found (*p*-trend <0.05). No interaction between DII and HOMA-IR was observed in terms of the risk of depression, and HOMA-IR did not find to play a mediating role in the association between DII and depression. Similar results were obtained for the association between E-DII and depression.

**Conclusion:**

Our results suggest that a higher pro-inflammatory diet increases the risk of depression in U.S. adults, while there was no evidence of a multiplicative effect of DII or E-DII and HOMA-IR on disease risk, nor of a mediating effect of HOMA-IR.

## 1. Introduction

Depression is one of the most common mental illnesses and a leading cause of disability worldwide, which has become an increasingly serious public health problem (1, 2). At present, the exact etiology and mechanism of depression are not fully understood, but it is certain that inflammation plays a key role in the occurrence and development of depression (1). Elevated levels of inflammatory factors such as C-reactive protein (CRP) and interleukin 6 (IL-6) may promote the development of depression (3). At the same time, different dietary patterns may show different inflammatory states (4). Previous studies have shown that unhealthy dietary patterns can increase the blood concentration of inflammatory markers such as CRP, complement component C3 and other cytokines (5). So, anti-inflammatory diet may be used as a potential prevention and intervention for depression (6, 7).

Dietary inflammatory index (DII) is developed to assess the overall inflammatory potential of an individual’s diet in a quantitative manner, avoiding the problems of single nutrient-disease studies that struggle to capture the overall impact of diet on health (8). Although several studies have analyzed the association between dietary inflammation and depression, the results have been inconsistent (9, 10). The differences in the results of these studies may be attributed to potential factors which have not been fully considered, such as insulin resistance (IR). IR is a pathological condition defined as an impaired response to insulin stimulation in peripheral tissues, resulting in elevated peripheral insulin levels (11). Existing evidence shows that DII is positively associated with homeostatic model assessment of insulin resistance (HOMA-IR), in which a more pro-inflammatory diet can increase the probability of IR (8). Furthermore, IR is a known risk factor that is positively associated with depression (12, 13), and it has been considered a mediator of the increased risk of depression observed in various clinical populations (14). Therefore, IR may play an important role between dietary inflammation and depression.

At present, there is currently no effective treatment to cure or prevent depression, and it is crucial to have a comprehensive understanding of the risk factors for depression and to improve prevention and treatment in a targeted manner (15). Inflammation and IR are inextricably linked to depression respectively, and inflammation and insulin resistance are closely related processes. However, the relationship between these three is not yet clear, unraveling the association will help to determine the prevention and treatment strategies of the disease. Therefore, we evaluated the association between dietary inflammatory potential and depression in adult population based on the National Health and Nutrition Examination Survey (NHANES), and analyzed the mediating role of IR in this relationship.

## 2. Methods

### 2.1. Participants and study design

**Figure 1:**
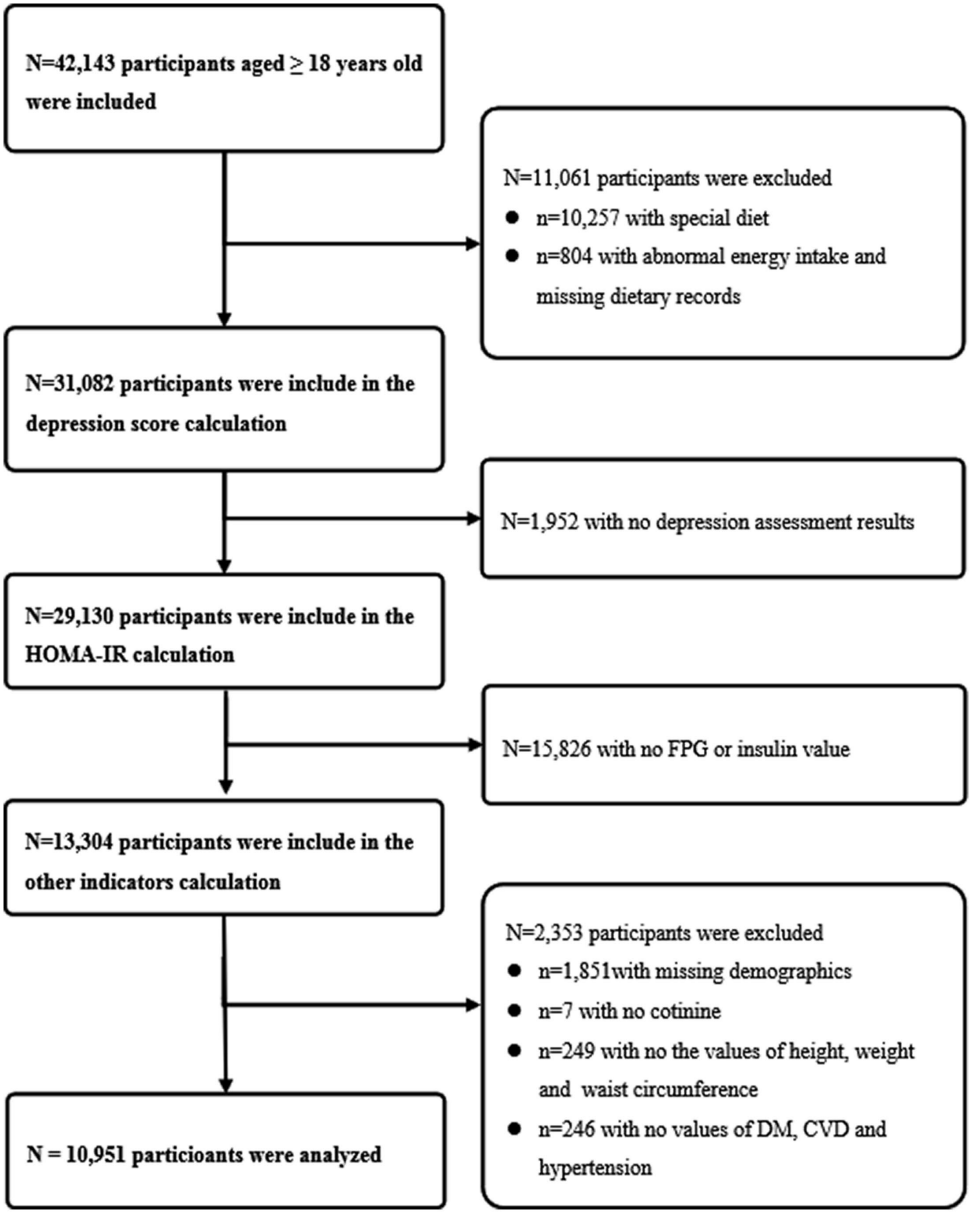
Flow chart of the study design. The NHANES 2005–2018 dataset included a total of 42,143 adult participants (aged ≥18 years). First, 10,257 participants with special dietary, 804 participants with abnormal energy intake (daily energy intake ≤500 kcal or ≥ 5,000 kcal) or missing dietary records were excluded. Second, 1,952 participants without depression assessment results were excluded. Third, 15,826 participants without fasting plasma glucose or insulin value were excluded. Fourth, 1,851 participants without demographics (gender, education level, race, marital status and poverty index ratio), 7 participants without serum cotinine data, 249 participants without physical examination data (height, weight and waist circumference) or 246 participants without medical history data (diabetes mellitus, cardiovascular diseases and hypertension) were excluded. Finally, 10,951 participants were included in the final analysis.

We used seven cycles of the NHANES (2005–2018), a population-based, nationwide cross-sectional survey. NHANES recruited a representative sample of civilian, community dwelling members of the US population using a complex, multistage probability design. Details of the study design and data collection have been previously described.^1^ Among 42,143 adult participants (aged ≥18 years) were included, and we excluded (1) special dietary, abnormal energy intake (daily energy intake ≤500 kcal or ≥5,000 kcal) or missing dietary records (*n* = 11,061); (2) without depression assessment results (*n* = 1,952); (3) without fasting plasma glucose (FPG) or insulin value (*n* = 15,826); (4) without demographics [gender, education level, race, marital status and poverty index ratio (PIR)] or serum cotinine data or physical examination data [height, weight and waist circumference (WC)] or medical history data [diabetes mellitus (DM), cardiovascular diseases (CVD) and hypertension] (*n* = 2,353). Finally, 10,951 participants were obtained for the statistical analysis (Figure 1).

### 2.2. Dietary inflammatory index

The overall inflammatory potential of individual diets was assessed using the DII. The standard mean and standard deviation of each dietary ingredient or nutrient parameter are available through the world database. For each dietary ingredient or nutrient, create a Z-score by subtracting the individual’s estimated intake from the standard average. It is then divided by the world standard deviation and converted to a distribution centred at 0 and bounded between −1 and +1 (16). Then, this value is multiplied by the corresponding inflammatory effect score, and then all dietary ingredient or nutrient parameters are summed together to obtain the overall DII score for the individual’s diet. DII scores range from negative tail to positive tail, more negative values indicate anti-inflammatory properties and corrected scores indicate proinflammatory properties (17). Considering the effect of total energy intake, the energy-adjusted dietary inflammatory index (E-DII) based on DII was created (18–20). All nutrient data in the dietary records and the global dietary intake database were converted to values per 1,000 kcal by dividing these data by the energy intake from the diet and multiplying by 1,000 (18–20).

Dietary intake was assessed from a single 24-h dietary recall in NHANES. It contains 27 dietary ingredient or nutrient parameters used to calculate the E-DII or DII score, including carbohydrates, protein, fat, cholesterol, (saturated, monounsaturated and polyunsaturated) fatty acids, omega-3 and omega-6 polyunsaturated fatty acids, vitamins (A, B1, B2, B6, B12, C, D, E), niacin, iron, magnesium, zinc, selenium, folic acid, beta carotene, alcohol, fiber and caffeine (21).

### 2.3. Depression

Depressive symptoms were evaluated using the 9-item Patient Health Questionnaire (PHQ-9), which is a validated 9-item screening instrument that asks about the frequency of depressive symptoms over the past 2 weeks. Each of the nine items consists of four questions, including “not at all,” “several days,” “more than half the day,” and “nearly every day,” and is scored from 0 to 3. Total scores of PHQ-9 range from 0 to 27. The higher the score, the more severe the depressive symptoms. The PHQ-9 scores of 10 or higher was used as the cut-off point to identify depression which had a sensitivity of 88% and a specificity of 88% for the diagnosis of major depression (22).

### 2.4. Homeostatic model assessment-insulin resistance

The HOMA-IR was used to assess the level of IR in individuals. HOMA-IR is the product of fasting insulin (μU/mL) and fasting blood glucose (mmol/L) divided by 22.5 (23). The value of HOMA-IR of normal individuals is 1, and the higher the value of HOMA-IR is, the stronger the resistance of individuals to insulin is (23).

### 2.5. Study covariates

The included covariates included gender (female or male), education level (less than high school, completed high school and more than high school), race (non-Hispanic white, non-Hispanic black, Mexican American, other Hispanic and other), marital status (never married, married, separated and divorced, widowed), age, PIR, CVD, DM, hypertension, body mass index (BMI), WC, energy intake, alcohol use, and serum cotinine.

### 2.6. Statistical analysis

The recommended weighting method was used to analyze NHANES data. Sample characteristics were reported as weighted mean ± standard deviation for normally distributed continuous variables, weighted medians (interquartile ranges) for nonnormally distributed continuous variables, and weighted proportions for categorical variables. Differences in characteristics between depressed and non- depressed groups were compared using Student’s *t*-test (continuous variables with normal distribution), Wilcoxon rank sum test (continuous variables with non-normal distribution) or *χ*^2^ test (categorical variables). One-way ANOVA tests were utilized to evaluate between-group differences of distributions across quartiles of E-DII or DII. Three statistical models were fitted and a logistic regression was used to estimate odds ratio (OR) and 95% confidence interval (CI) of depression in relation to quartiles of E-DII. Model 1 did not adjust any covariates; Model 2 adjusted for gender, education level, race, marital status, age, PIR, CVD, DM, hypertension, BMI, WC, energy intake, alcohol use and serum cotinine. Model 3 is adjusted for all covariates included in Model 2 as well as HOMA-IR. In order to assess the dose–response relationship between E-DII or DII and depression in all three models, using a restricted cubic spline model with three knots located at the 25th, 50th and 75th percentiles of E-DII levels.

Stratified analysis of HOMA-IR (quartile) was performed under Model 1 and Model 2 to assess the potential regulatory role of IR, and analyzed the effect of the interaction between E-DII or DII and HOMA-IR on depression. The mediation model and the outcome model used linear regression and logistic regression, respectively, to assess the direct and indirect effects of HOMA-IR between E-DII or DII and depression. In addition, we performed a sensitivity analysis. The interval of E-DII or DII was re-divided in tertiles, excluding the influence of different division methods of E-DII or DII interval on the results. All statistical analysis was performed by R 4.1.2 (R Foundation for Statistical Computing, Vienna, Austria). Two-sided *p* < 0.05 was considered statistically significant.

## 3. Results

### 3.1. Characteristics of participants

10,091 participants were included in the non-depressed group, and the remaining 860 participants were included in the depressed group. Compared with non-depressed, depressed participants had higher proportions of female, education level less than high school, non-Hispanic black, separated and divorced and serum cotinine (*p* < 0.05). Meanwhile, depressed participants had higher levels of physical examination indexes (BMI and WC), sugar metabolism measurements (FPG, insulin and HOMA-IR), and the basic diseases (CVD, DM and hypertension) (*p* < 0.05). Furthermore, the participants with depression had a higher level of DII or E-DII. The general characteristics of the study population were shown in Table 1.

**Table 1:** Characteristics of the study population.

Variable	Depression		*χ^2^*/*t*	*p* value
	No (*n* = 10,091)	Yes (*n* = 860)		
Age	47.63 ± 17.11	47.39 ± 15.27	−0.32	0.751
Gender			19.18	<0.001
Female	4,734 (0.48)	523 (0.60)		
Male	5,357 (0.52)	337 (0.40)		
Education level			18.23	<0.001
Less than high school	2,280 (0.14)	295 (0.24)		
Completed high school	2,352 (0.24)	237 (0.32)		
More than high school	5,459 (0.62)	328 (0.44)		
Race			3.26	0.024
Non-Hispanic white	4,629 (0.70)	385 (0.64)		
Non-Hispanic black	1,998 (0.10)	190 (0.13)		
Mexican American	1,543 (0.08)	128 (0.08)		
Other Hispanic and other	1,921 (0.12)	157 (0.14)		
Marital status			18.00	<0.001
Never married	2,611 (0.26)	265 (0.31)		
Married	5,410 (0.56)	295 (0.37)		
Separated and divorced	1,368 (0.13)	222 (0.25)		
Widowed	702 (0.05)	78 (0.07)		
PIR	3.08 ± 1.63	1.98 ± 1.53	−14.81	<0.001
BMI	28.41 ± 6.51	29.91 ± 7.63	4.90	<0.001
WC	98.07 ± 16.22	100.98 ± 17.29	3.97	<0.001
Serum cotinine	59.40 ± 129.43	123.97 ± 167.65	7.33	<0.001
Alcohol use	10.99 ± 25.62	10.85 ± 31.60	−0.08	0.937
Energy intake	2,169.33 ± 849.57	2,018.33 ± 906.21	−2.99	0.003
FPG	5.82 ± 1.50	6.08 ± 2.10	2.74	0.007
HOMA-IR	3.39 ± 5.13	4.36 ± 6.15	3.48	0.001
Insulin	12.25 ± 13.32	14.49 ± 13.43	3.47	0.001
DII	0.99 ± 1.94	1.75 ± 1.89	8.34	<0.001
E-DII	0.96 ± 1.79	1.54 ± 1.66	6.26	<0.001
CVD			19.15	<0.001
No	9,192 (0.93)	699 (0.85)		
Yes	899 (0.07)	161 (0.15)		
DM			8.24	0.005
No	8,322 (0.87)	654 (0.82)		
Yes	1,769 (0.13)	206 (0.18)		
Hypertension			14.23	<0.001
No	6,637 (0.69)	464 (0.57)		
Yes	3,454 (0.31)	396 (0.43)		
Quartiles of HOMA-IR			7.60	<0.001
Q1	2,572 (0.28)	166 (0.22)		
Q2	2,537 (0.26)	201 (0.24)		
Q3	2,545 (0.24)	192 (0.21)		
Q4	2,437 (0.22)	301 (0.33)		
Quartiles of DII			23.13	<0.001
Q1	2,593 (0.28)	145 (0.17)		
Q2	2,554 (0.26)	184 (0.18)		
Q3	2,523 (0.24)	214 (0.28)		
Q4	2,421 (0.22)	317 (0.37)		
Quartiles of E-DII			9.13	
Q1	2,593 (0.26)	145 (0.17)		<0.001
Q2	2,536 (0.25)	202 (0.22)		
Q3	2,525 (0.25)	212 (0.26)		
Q4	2,437 (0.23)	301 (0.34)		

Q1, first quartile; Q2, second quartile; Q3, third quartile; Q4, fourth quartile; BMI, body mass index; DII, dietary inflammatory index; E-DII, energy-adjusted dietary inflammatory index; HOMA-IR, homeostatic model assessment for insulin resistance; PIR, poverty index ratio; WC, waist circumference; CVD, cardiovascular disease; DM, diabetes mellitus.

### 3.2. Characteristics of the participants according to the quartiles of DII or E-DII

The characteristics of participants by DII quartiles were shown in Table 2. The higher the DII scores, the higher the BMI, waist circumference, serum cotinine, energy intake, FPG, insulin and HOMA-IR (*p* < 0.05). In contrast, age and PIR decreased gradually (*p* < 0.05). Simultaneously, compared with first quartile, those participants who in the second quartile to the fourth quartile of DII group had higher proportions of male, non-Hispanic black, never married, and the risk of depression (*p* < 0.05). However, according to the E-DII quartiles, the higher E-DII scores, the higher proportions of CVD, DM, Hypertension were (*p* < 0.05), and age was no longer significantly different (*p* > 0.05). The remaining characteristics of the participants according to the quartiles of E-DII were similar to those of DII (Supplementary Table S1).

**Table 2:** Characteristics of the participants according to the quartiles of DII.

Variable	Quartiles of DII				*χ^2^*/*F*	*p-*value
	Q1	Q2	Q3	Q4		
Age	51.78 ± 17.19	48.51 ± 17.03	45.98 ± 16.57	44.04 ± 16.12	108.84	<0.001
Gender					14.1	<0.001
Female	1,491 (0.56)	1,294 (0.48)	1,208 (0.46)	1,264 (0.47)		
Male	1,247 (0.44)	1,444 (0.52)	1,529 (0.54)	1,474 (0.53)		
Education level					17.52	<0.001
Less than high school	554 (0.11)	675 (0.15)	620 (0.15)	726 (0.19)		
Completed high school	509 (0.18)	562 (0.21)	741 (0.29)	777 (0.29)		
More than high school	1,675 (0.70)	1,501 (0.64)	1,376 (0.56)	1,235 (0.51)		
Race					16.81	<0.001
Non-Hispanic white	1,219 (0.70)	1,263 (0.71)	1,218 (0.69)	1,314 (0.69)		
Non-Hispanic black	405 (0.07)	471 (0.08)	604 (0.11)	708 (0.14)		
Mexican American	411 (0.07)	471 (0.09)	431 (0.08)	358 (0.07)		
Other Hispanic and other	703 (0.16)	533 (0.12)	484 (0.11)	358 (0.09)		
Marital status					7.65	<0.001
Never married	553 (0.21)	647 (0.24)	788 (0.28)	888 (0.33)		
Married	1,598 (0.62)	1,451 (0.55)	1,391(0.52)	1,265 (0.49)		
Separated and divorced	351 (0.10)	424 (0.15)	379 (0.15)	436 (0.14)		
Widowed	236 (0.07)	216 (0.06)	179 (0.05)	149 (0.04)		
PIR	3.34 ± 1.62	3.03 ± 1.66	2.91 ± 1.6	2.69 ± 1.62	75.53	<0.001
BMI	27.26 ± 5.69	28.09 ± 6.19	29.29 ± 7.00	29.49 ± 7.20	70.50	<0.001
WC	95.19 ± 14.91	97.43 ± 15.51	100.17 ± 16.85	100.43 ± 17.35	64.54	<0.001
Serum cotinine	28.42 ± 91.82	55.36 ± 126.54	73.37 ± 138.01	100.66 ± 159.84	145.73	<0.001
Alcohol use	8.12 ± 17.00	11.11 ± 23.30	13.36 ± 29.66	11.35 ± 31.84	19.03	<0.001
Energy intake	1,908.16 ± 728.84	2,132.59 ± 839.11	2,279.84 ± 866.23	2,320.1 ± 915.64	134.89	<0.001
FPG	5.75 ± 1.43	5.91 ± 1.77	5.85 ± 1.44	5.84 ± 1.56	4.71	0.003
Insulin	10.17 ± 8.84	12.19 ± 16.22	13.78 ± 14.45	13.54 ± 12.34	42.87	<0.001
HOMA-IR	2.74 ± 3.09	3.55 ± 7.10	3.82 ± 4.87	3.75 ± 4.90	25.39	<0.001
CVD					0.77	0.514
No	2,484 (0.92)	2,454 (0.91)	2,486 (0.93)	2,467 (0.92)		
Yes	254 (0.08)	284 (0.09)	251 (0.07)	271 (0.08)		
DM					0.83	0.48
No	2,235 (0.87)	2,187 (0.86)	2,243 (0.86)	2,311 (0.88)		
Yes	503 (0.13)	551 (0.14)	494 (0.14)	427 (0.12)		
Hypertension					1.17	0.324
No	1,718 (0.67)	1,762 (0.68)	1,814 (0.69)	1,807 (0.67)		
Yes	1,020 (0.33)	976 (0.32)	923 (0.31)	931 (0.33)		
Quartile groups of HOMA-IR					13.03	<0.001
Q1	824 (0.35)	663 (0.27)	628 (0.25)	623 (0.23)		
Q2	750 (0.27)	712 (0.28)	640 (0.25)	636 (0.24)		
Q3	639 (0.21)	694 (0.24)	699 (0.24)	705 (0.26)		
Q4	525 (0.17)	669 (0.21)	770 (0.26)	774 (0.28)		
Depression					9.13	<0.001
No	2,593 (0.95)	2,536 (0.94)	2,525 (0.92)	2,437 (0.90)		
Yes	145 (0.05)	202 (0.06)	212 (0.08)	301 (0.10)		

Q1, first quartile; Q2, second quartile; Q3, third quartile; Q4, fourth quartile; BMI, body mass index; E-DII, energy-adjusted dietary inflammatory index; HOMA-IR, homeostatic model assessment for insulin resistance; PIR, poverty index ratio; WC, waist circumference; CVD, cardiovascular disease; DM, diabetes mellitus.

### 3.3. Association between DII or E-DII and depression

**Figure 2:**
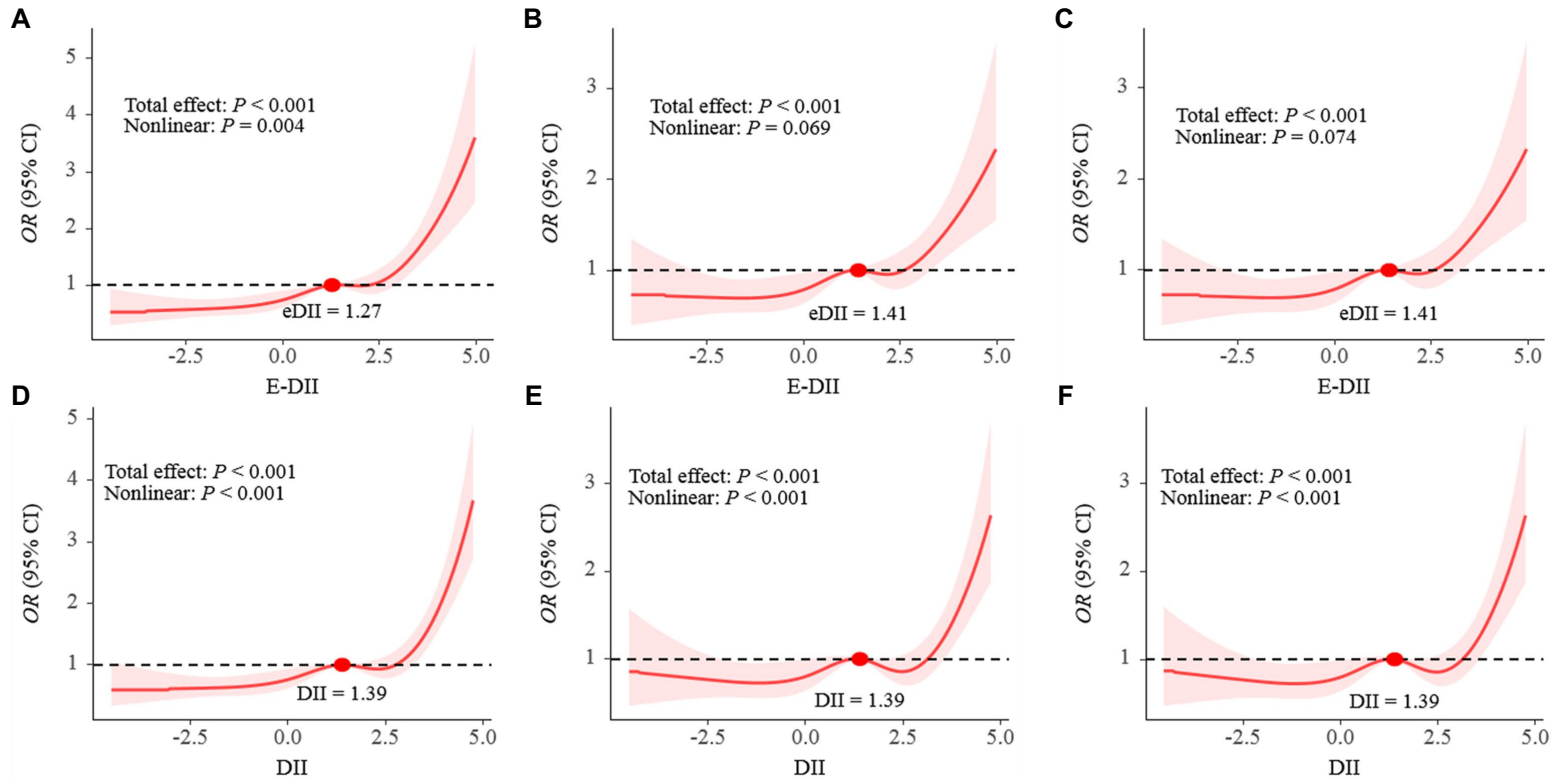
The dose–response relationships of E-DII **(A–C)** and DII **(D–F)** with depression in all participants. Results were from restricted cubic spline models; **A** and **D** did not adjust any covariates; **B** and **E** were adjusted for gender, education, race, marital status, age, PIR, CVD, DM, hypertension, BMI, WC, energy intake, alcohol use, serum cotinine; **C** and **F** was adjusted for gender, education, race, marital status, age, PIR, CVD, DM, hypertension, BMI, WC, energy intake, alcohol use, serum cotinine, HOMA-IR.

As shown in Table 3, DII was positively correlated with the risk of depression in the third quartile [*OR*: 1.89 (95% *CI*: 1.33–2.69)] to the fourth quartile [*OR*: 2.85 (95% *CI*: 2.13–3.81)] compared with the first quartile in Model 1. DII was also positively correlated with the risk of depression in the fourth quartile compared with the first quartile in Model 2 and Model 3 [*OR*:1.82 (95% *CI*: 1.28–2.58)]. Moreover, a significant dose–response relationship was found in all three models (*p*-trend <0.001). An analysis with DII to increase 1-SD yielded similar results in Model 1 [*OR*: 1.53 (95% *CI*: 1.37–1.71)], Model 2 and Model 3 [*OR*: 1.26 (95% *CI*: 1.10–1.45)]. Meanwhile, the association between E-DII and depression had the same characteristics. As shown in Figure 2, the spline variable confirmed that DII in all three models and E-DII in Model 1 were significant non-linearly associated with the risk of depression (*P*_nonlinear_ < 0.05). While there was a linear dose–response relationship between E-DII score and the risk of depression in Model 2 (*P*_nonlinear_ = 0.069) and Model 3 (*P*_nonlinear_ = 0.074).

**Table 3:** Risk of depression according to quartile groups of DII or E-DII.

	Model 1	Model 2	Model 3
	OR (95% CI)	OR (95% CI)	OR (95% CI)
**Quartiles of DII**			
Q1	Reference		
Q2	1.11 (0.79–1.55)	0.95 (0.68–1.32)	0.95 (0.68–1.32)
Q3	1.89 (1.33–2.69)	1.41 (0.95–2.11)	1.41 (0.95–2.11)
Q4	2.85 (2.13–3.81)	1.82 (1.28–2.58)	1.82 (1.28–2.58)
*p*-trend	<0.001	<0.001	<0.001
For 1-SD increase	1.53 (1.37–1.71)	1.26 (1.10–1.45)	1.26 (1.10–1.45)
**Quartiles of E-DII**			
Q1	Reference		
Q2	1.34 (0.89–2.01)	1.12 (0.75–1.66)	1.11 (0.75–1.65)
Q3	1.60 (1.12–2.29)	1.26 (0.89–1.79)	1.26 (0.88–1.79)
Q4	2.25 (1.60–3.17)	1.54 (1.10–2.17)	1.54 (1.09–2.17)
*p*-trend	<0.001	<0.001	<0.001
For 1-SD increase	1.42 (1.26–1.61)	1.23 (1.09–1.40)	1.23 (1.09–1.40)

Q1, first quartile; Q2, second quartile; Q3, third quartile; Q4, fourth quartile; SD, standard deviation; OR, odds ratio; CI, confidence interval; E-DII, energy-adjusted dietary inflammatory index; DII, dietary inflammatory index. Model 1: Did not adjust any covariates. Model 2: Adjusted for gender, education, race, marital status, age, PIR, CVD, DM, hypertension, BMI, WC, energy intake, alcohol use, and serum cotinine. Model 3: Adjusted for gender, education, race, marital status, age, PIR, CVD, DM, hypertension, BMI, WC, energy intake, alcohol use, serum cotinine, HOMA-IR.

### 3.4. DII or E-DII and depression risk stratified by HOMA-IR category

As shown in Table 4, when stratified by HOMA-IR category, the risk of DII and depression was mainly reflected in fourth quartile of DII except people with 2.42 ≤HOMA-IR <4.20 in Model 1 [the *OR*s (95% *CI*s) were 5.07 (2.67–9.62), 4.04 (2.21–7.38), 1.67 (1.01–2.77), respectively]. And in Model 2, the risk of DII and depression was mainly reflected in the fourth quartile of DII in the population with HOMA-IR <2.42 [the *OR*s (95% *CI*s) were 2.48 (1.15–5.36), 2.04 (1.03–4.02), respectively]. The risk of E-DII and depression had the same characteristics. Besides, there was no interaction between DII or E-DII and depression on depression risk (*P*_interaction_ > 0.05).

**Table 4:** Risk of depression by quartile of DII or E-DII stratified according to quartiles of HOMA-IR.

	Model 1	Model 2
	OR (95% CI)	OR (95% CI)
**Quartiles of DII**		
*0.03 ≤ HOMA-IR < 1.46*		
Q1	Reference	
Q2	1.51 (0.69–3.33)	1.16 (0.53–2.57)
Q3	3.15 (1.59–6.23)	1.80 (0.87–3.75)
Q4	5.07 (2.67–9.62)	2.48 (1.15–5.36)
*1.46 ≤ HOMA-IR < 2.42*		
Q1	Reference	
Q2	1.19 (0.68–2.08)	0.94 (0.49–1.79)
Q3	1.63 (0.74–3.58)	1.22 (0.47–3.14)
Q4	4.04 (2.21–7.38)	2.36 (1.13–4.90)
*2.42 ≤ HOMA-IR < 4.20*		
Q1	Reference	
Q2	0.81 (0.39–1.71)	0.77 (0.35–1.73)
Q3	1.07 (0.51–2.24)	1.10 (0.43–2.79)
Q4	1.82 (0.87–3.81)	1.80 (0.71–4.55)
*HOMA-IR ≥ 4.20*		
Q1	Reference	
Q2	0.96 (0.54–1.70)	0.86 (0.48–1.55)
Q3	1.81 (1.06–3.08)	1.53 (0.81–2.90)
Q4	1.67 (1.01–2.77)	1.17 (0.54–2.54)
*P* ^a^_interaction_	0.862	0.661
**Quartiles of E-DII**		
*0.03 ≤ HOMA-IR < 1.46*		
Q1	Reference	
Q2	1.74 (0.82–3.67)	1.16 (0.53–2.53)
Q3	2.00 (0.96–4.18)	1.23 (0.55–2.76)
Q4	2.86 (1.43–5.73)	1.43 (0.68–3.00)
*1.46 ≤ HOMA-IR < 2.42*		
Q1	Reference	
Q2	1.64 (0.71–3.78)	1.68 (0.86–3.32)
Q3	1.46 (0.61–3.49)	1.34 (0.65–2.79)
Q4	2.41 (1.03–5.61)	2.04 (1.03–4.02)
*2.42 ≤ HOMA-IR < 4.20*		
Q1	Reference	
Q2	0.50 (0.26–0.97)	0.46 (0.22–0.94)
Q3	1.12 (0.56–2.24)	0.97 (0.46–2.06)
Q4	2.17 (1.14–4.14)	1.54 (0.72–3.31)
*HOMA-IR ≥ 4.20*		
Q1	Reference	
Q2	1.36 (0.76–2.44)	1.23 (0.66–2.28)
Q3	1.43 (0.81–2.52)	1.40 (0.75–2.62)
Q4	1.40 (0.80–2.43)	1.29 (0.70–2.37)
*P* ^b^_interaction_	0.536	0.629

Q1, first quartile; Q2, second quartile; Q3, third quartile; Q4, fourth quartile; OR, odds ratio; CI, confidence interval; E-DII, energy-adjusted dietary inflammatory index; DII, dietary inflammatory index; HOMA-IR, homeostatic model assessment for insulin resistance. *P*^a^_interaction_ is the value of interaction between DII and HOMA-IR on depression; *P*^b^_interaction_ is the value of interaction between E-DII and HOMA-IR on depression; E-DII is empirical dietary inflammatory index; Model 1: Did not adjust any covariates; Model 2: Adjusted for gender, education, race, marital status, age, PIR, CVD, DM, hypertension, BMI, WC, energy intake, alcohol use, and serum cotinine.

### 3.5. Mediating role of HOMA-IR

**Figure 3:**
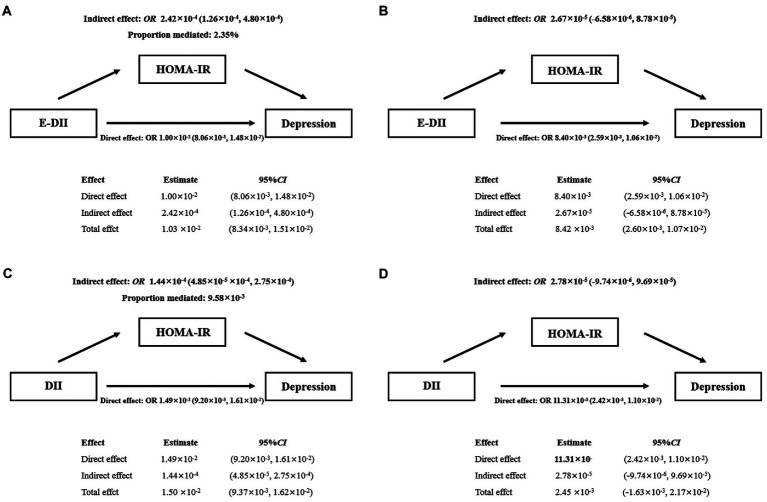
Mediating effect of HOMA-IR between E-DII **(A,B)** or DII **(C,D)** and depression. The 95% CI of these estimates was computed using the bootstrap method (1,000 samples); **A** and **C** did not adjust any covariates; **B** and **D** were adjusted for gender, education, race, marital status, age, PIR, CVD, DM, hypertension, BMI, WC, energy intake, alcohol use, serum cotinine.

As shown in Figure 3, increased E-DII was associated with an increased risk of depression, and the effect (2.35%) can be explained by a significant indirect effect of HOMA-IR (*OR*: 2.42 × 10^−4^, 95% *CI*: 1.26 × 10^−4^ − 4.80 × 10^−4^) (Figure 3A). After adjusting for covariates, the indirect effect was not statistically significant (*OR*: 2.67 × 10^−5^, 95% *CI*: −6.58 × 10^−6^ − 8.78 × 10^−5^) (Figure 3B). The data were analyzed using the DII quartiles, and the same result was reached (Figures 3C,D).

### 3.6. Mediating role of HOMA-IR

According to the DII tertiles, the *ORs* (95% *CI*s) for the depression were 1.42 (1.00–2.00) and 1.61 (1.17–2.21) for the second and third tertile in Model 3 (*p*-trend <0.05). And, E-DII was positively correlated with the risk of depression in the third tertile compared with the first tertile [*OR*:1.45 (95% *CI*:1.13–1.86)] (Supplementary Table S2). The risk of DII and depression was found in the second and third tertile of DII except people with HOMA-IR ≥ 4.20 in Model 1. In addition, the *ORs* and 95% *CIs* for the risk of DII and depression was mainly reflected in third tertile of DII with 0.03 ≤ HOMA-IR < 1.46 and 1.46 ≤ HOMA-IR < 2.42 in Model 2 were 1.90 (1.04–3.49) and 1.91 (1.03–3.55), respectively. Meanwhile, the risk of E-DII and depression was mainly reflected in third tertile of E-DII with 0.03 ≤ HOMA-IR < 1.46 and 2.42 ≤ HOMA-IR < 4.20 in Model 1 [the *OR*s (95% *CI*s) were 2.50 (1.44–4.35), 2.44 (1.34–4.46), respectively] (Supplementary Table S3).

## 4. Discussion

In this large cross-sectional study to investigate how DII or E-DII and IR contribute to depression risk, we found that DII or E-DII was an independent risk factor for depression, and there was a positive non-linear relationship between the DII and the risk of depression. While, there was a linear dose–response relationship between E-DII score and the risk of depression in Model 2 and Model 3. In addition, IR-related indicators (insulin level, FPG and HOMA-IR) were higher in depressed patients, and these indicators gradually increased as the score of E-DII or DII increased. In models not adjusted for covariates, HOMA-IR had a weak mediating effect on E-DII or the association between DII and depression. After adjusting the model, the mediating effect of HOMA-IR between E-DII or DII and depression disappeared. Moreover, there was no evidence of significant interactions on the multiplicative scale between E-DII or DII and HOMA-IR on the risk of depression.

Our results showed that both DII and E-DII scored higher in the depressed group compared to the non-depressed group (Table 1). The higher the DII or E-DII score, the higher the risk of depression (Table 3). This was consistent with some studies. Both in the North West Adelaide Health Study cohort (10) and Chen et al. (24) study found that patients with depression had a higher dietary inflammatory potential compared with the general population control group, and those with higher DII or E-DII scores had higher odds of depression. However, the sample sizes of their researches were relatively small, and the representativeness of the sample still needed to be further improved. In addition, their studies only analyzed the association between DII or E-DII and depression alone, and only considered one division method (using quartiles or tertiles) for DII or E-DII. In this way, the false positive or false negative results caused by the interference of the division interval of DII or E-DII could not be ruled out, so the robustness of the data results still needed to be further improved. In contrast, our study not only used data from a large, nationally representative population sample, but also analyzed the correlation between DII and E-DII and depression, and the sensitivity analysis was made. This greatly improves the representativeness of the sample and the robustness of the data results.

But it was also inconsistent with the results of some studies. The DII was not associated with incident depressive symptoms in the Suppl émentation en Vitamines et Min éraux Antioxydants (SU.VI.MAX) cohort (25). Meanwhile, in the Korea National Health and Nutrition Examination Survey (KNHANES) 2014–2017, no association between E-DII and depression/depressive symptoms was found among adults in other parts of South Korea except the Capital area, Chungcheong-do and Jeju-do (26). These are somewhat different from our findings, and we speculate that the possible reasons are as follows. First, it may be affected by the different ethnicity, dietary patterns and living area of the study population. Our study subjects are from the United States, while the subjects included in SU.VI.MAX cohort are from France, and the KNHANES is included in the South Korean population. Second, affected by the age difference of the study population. Adults (≥18 years) were included in our study, while 35–60 years were included in SU.VI.MAX cohort and aged ≥19 years were included in KNHANES.

In our study, FBG, insulin levels and HOMA-IR were higher in depressed patients compared with controls (Table 1), and with the increase of DII or E-DII, FBG, insulin levels and HOMA-IR also gradually increased (Table 2). A large meta-analysis found increased insulin levels and HOMA-IR in patients with depression (12), which was consistent with our findings. Some of studies have confirmed the correlation between IR and depression. Kan et al. found that, a small but significant cross-sectional association was observed between depression and IR (27). Studies from a large United Kingdom birth cohort showed that IR was positively associated with depression (28). According to a cross-sectional study in Korea, after adjusting covariates, increased IR was weakly associated with greater depressive symptoms (adjusted *OR* = 1.01, 95% *CI* = 1.0001–1.03) (29). So far, the relationship between IR and DII or E-DII has not yet reached a consensus. In an Iranian adult cohort, no significant associations were observed between DII and risk of FPG (*p* = 0.07), fasting insulin (*p* = 0.07) or HOMA-IR (*p* = 0.08) (30). However, more research supported that IR or IR-related indicators were closely related to DII or E-DII (8, 30, 31). Our study also confirmed this point of view, providing new supporting evidence for the association between IR and dietary inflammation. Existing studies have shown that the key metabolic changes caused by inflammation are an important factor leading to insulin receptor dysfunction, which further has a major impact on the metabolism of the brain and promotes the occurrence and development of mental diseases including depression (32). However, our results showed that the association of DII or E-DII with depression was significant regardless of adjustment for HOMA-IR (Table 3), suggesting that the association of DII or E-DII with depression may be independent of IR. Additionally, in the stratified model, there was only an increased risk of depression in the Q3 or Q4 range of DII or E-DII in some intervals of HOMA-IR, and no interaction between DII or E-DII and HOMA-IR was found to reach statistical significance (Table 4). This means that HOMA-IR and DII or E-DII may have independent effects on depression. If there are interactions between HOMA-IR and E-DII or DII, they are likely to be small, undetectable by conventional methods, or influenced by other unknown factors. In the mediation analysis, HOMA-IR only played a weak mediating effect in the unadjusted model (Figures 3A,C), and the mediating effect did not exist after multivariate adjustment (Figures 3B,D). This suggests that the effect of HOMA-IR on the association between DII or E-DII and depression may be influenced by other variables. Future studies could select potential mediators from the adjusted covariates and investigate their role in the association of DII or E-DII with depression.

The strengths of this study include the use of large sample data with national representation, and the simultaneous exploration of the association between DII or E-DII and depression, as well as the role of IR in it, through multiple analytical methods. Furthermore, we found consistency in sensitivity analyses. Our study elucidated that DII or E-DII was independently associated with depression risk, that IR and DII or E-DII had no synergistic effect on disease risk, and that IR did not mediate this association, which had important clinical and public health implications. Since diet is a relatively easy and modifiable factor, it may be possible to prevent depression and reduce depressive symptoms by limiting pro-inflammatory diets or encouraging anti-inflammatory diets. However, we have to admit that our study still has some limitations. First, the E-DII or DII comprised 45 food parameters, but only 27 food parameters were included in the calculations and the estimates of the potential of dietary inflammation might have deviations. But this is common because a complete assessment of all food intakes is difficult and DII scores can be calculated using >20 items from the list of required food parameters (33). Second, the PHQ-9 parental questionnaire was used to assess depressive symptoms, which was only a screening tool, not an exact diagnostic tool. However, its sensitivity and specificity are high, and the misclassification may be relatively few (34). Third, the results of the present study were based on American adults, which the true state of other populations might not be accurately reflected. Fourthly, this study was a cross-sectional study, which leaded to a lack of evidence of the cause and effect. Further prospective field intervention studies should be conducted in the future to provide stronger evidence for the relationship between DII or E-DII and depression.

In conclusion, increased E-DII or DII promoted elevated levels of indicators related to IR and were independently associated with the risk of depression in U.S. adults. There was no evidence of a multiplicative effect of E-DII or of DII and HOMA-IR on disease risk, nor of a mediating effect of HOMA-IR.

## Data availability statement

The original contributions presented in the study are included in the article/Supplementary material, further inquiries can be directed to the corresponding author/s.

## Ethics statement

The NHANES protocols were approved by the ethics review board and included the written informed consent of all participants, following the principles of the Declaration of Helsinki.

## Author contributions

Study conception and design by WH, JH, and YD. Data screening, sorting and analysis were performed by WH, LL, JH, RH, DK, and HY. The first draft of the manuscript was written by LL. Critical revision of the manuscript for important intellectual content by WH, JH, and HY. Study supervision by HY and YD. All authors contributed to the article and approved the submitted version.

## Funding

This work was supported by the Discipline Construction Project of Guangdong Medical University (4SG21276P); The Basic and Applied Basic Research Foundation of Guangdong Province Regional Joint Fund Project (The Key Project) (2020B1515120021); The Natural Science Foundation of Guangdong Basic and Applied Basic Research Fund (2022A1515012407) and Guangdong Province Traditional Chinese Medicine Science and Technology Research Project (20221209 and 20211215).

## Conflict of interest

The authors declare that the research was conducted in the absence of any commercial or financial relationships that could be construed as a potential conflict of interest.

## Publisher’s note

All claims expressed in this article are solely those of the authors and do not necessarily represent those of their affiliated organizations, or those of the publisher, the editors and the reviewers. Any product that may be evaluated in this article, or claim that may be made by its manufacturer, is not guaranteed or endorsed by the publisher.

## Footnotes

^1^ www.cdc.gov/nchs/nhanes/about_nhanes.htm
