# Supplementary material for: The association between dietary inflammation index and depression
Source: Front Psychiatry. 2023 Mar 23;14:1131802. doi: 10.3389/fpsyt.2023.1131802 (PMC10076670; doi:10.3389/fpsyt.2023.1131802)
Supplement: Supplementary file 1 [file Data_Sheet_1.docx]

Supplementary Material

The association between dietary inflammation index and depression

Ling Luo^1,†^, Jie Hu^2,†^, Ruixian Huang^1^, Danli Kong^1^, Haibing Yu^1,4,5^, Wei Hu^1,3,*^, Yuanlin Ding^1,*^

*** Correspondence:** Wei Hu: [huw1991@hotmail.com](mailto:huw1991@hotmail.com); Yuanlin Ding: gdmusbd@gdmu.edu.cn

**Table** **S1** Characteristics of the participants according to the quartiles of E-DII.

| Variable | Quartiles of E-DII | | | | *χ^2^*/*F* | *P* value |
| --- | --- | --- | --- | --- | --- | --- |
|  | Q1 [-5.21, -0.09] | Q2 (-0.09, 1.28] | Q3 (1.28, 2.37] | Q4 (2.37, 5.26] |  |  |
| Age | 48.02 ± 15.98 | 47.25 ± 16.95 | 47.52 ± 17.24 | 47.64 ± 17.90 | 1.03 | 0.376 |
| Gender |  |  |  |  | 88.40 | <0.001 |
| Female | 978 (0.36) | 1,206 (0.45) | 1,404 (0.54) | 1,669 (0.64) |  |  |
| Male | 1,760 (0.64) | 1,532 (0.55) | 1,333 (0.46) | 1,069 (0.36) |  |  |
| Education level |  |  |  |  | 24.49 | <0.001 |
| Less than high school | 478 (0.10) | 609 (0.15) | 686 (0.16) | 802 (0.20) |  |  |
| Completed high school | 545 (0.19) | 579 (0.20) | 702 (0.28) | 763 (0.31) |  |  |
| More than high school | 1,715 (0.71) | 1,550 (0.64) | 1,349 (0.56) | 1,173 (0.49) |  |  |
| Race |  |  |  |  | 9.02 | <0.001 |
| Non-Hispanic white | 1,325 (0.74) | 1,236 (0.69) | 1,229 (0.69) | 1,224 (0.67) |  |  |
| Non-Hispanic black | 382 (0.06) | 523 (0.10) | 617 (0.12) | 666 (0.14) |  |  |
| Mexican American | 468 (0.08) | 441 (0.09) | 404 (0.08) | 358 (0.07) |  |  |
| Other Hispanic and other | 563 (0.12) | 538 (0.13) | 487 (0.11) | 490 (0.12) |  |  |
| Marital status |  |  |  |  | 8.92 | <0.001 |
| Never married | 651 (0.25) | 716 (0.26) | 738 (0.26) | 771 (0.29) |  |  |
| Married | 1,617 (0.61) | 1,462 (0.55) | 1,398 (0.53) | 1,228 (0.47) |  |  |
| Separated and divorced | 331 (0.10) | 379 (0.14) | 409 (0.15) | 471 (0.16) |  |  |
| Widowed | 139 (0.04) | 181 (0.05) | 192 (0.06) | 268 (0.08) |  |  |
| PIR | 3.38 ± 1.64 | 3.12 ± 1.61 | 2.86 ± 1.62 | 2.54 ± 1.59 | 136.17 | <0.001 |
| BMI | 27.62 ± 5.97 | 28.47 ± 6.44 | 29.03 ± 6.89 | 29.13 ± 7.07 | 31.52 | <0.001 |
| WC | 96.94 ± 15.48 | 98.32 ± 16.42 | 99.17 ± 16.52 | 98.91 ± 16.84 | 10.70 | <0.001 |
| Serum cotinine | 41.01 ± 109.83 | 54.90 ± 126.03 | 72.57 ± 138.85 | 93.04 ± 154.52 | 78.39 | <0.001 |
| Alcohol use | 13.68 ± 26.07 | 13.90 ± 30.46 | 10.41 ± 26.68 | 5.06 ± 17.89 | 66.56 | <0.001 |
| Energy intake | 2,746.35 ± 857.55 | 2,335.45 ± 722.83 | 1,940.74 ± 646.09 | 1,482.22 ± 577.33 | 1,553.78 | <0.001 |
| FPG | 5.77 ± 1.44 | 5.83 ± 1.55 | 5.84 ± 1.51 | 5.93 ± 1.73 | 4.87 | 0.002 |
| Insulin | 11.37 ± 11.72 | 12.46 ± 16.19 | 13.19 ± 13.25 | 12.76 ± 11.53 | 9.83 | <0.001 |
| HOMA-IR | 3.15 ± 4.71 | 3.51 ± 6.55 | 3.61 ± 4.30 | 3.63 ± 4.97 | 5.29 | 0.001 |
| CVD |  |  |  |  | 7.93 | <0.001 |
| No | 2,546 (0.94) | 2,486 (0.92) | 2,470 (0.92) | 2,389 (0.90) |  |  |
| Yes | 192 (0.06) | 252 (0.08) | 267 (0.08) | 349 (0.10) |  |  |
| DM |  |  |  |  | 9.13 | <0.001 |
| No | 2,331 (0.90) | 2,264 (0.87) | 2,200 (0.85) | 2,181 (0.84) |  |  |
| Yes | 407 (0.10) | 474 (0.13) | 537 (0.15) | 557 (0.16) |  |  |
| Hypertension |  |  |  |  | 3.26 | 0.024 |
| No | 1,858 (0.70) | 1,806 (0.69) | 1,756 (0.68) | 1,681 (0.64) |  |  |
| Yes | 880 (0.30) | 932 (0.31) | 981 (0.32) | 1,057 (0.36) |  |  |
| Quartiles of HOMA-IR |  |  |  |  | 4.92 | <0.001 |
| Q1 | 785 (0.33) | 688 (0.28) | 623 (0.24) | 642 (0.25) |  |  |
| Q2 | 705 (0.26) | 690 (0.26) | 687 (0.26) | 656 (0.26) |  |  |
| Q3 | 670 (0.23) | 666 (0.23) | 697 (0.25) | 704 (0.25) |  |  |
| Q4 | 578 (0.18) | 694 (0.23) | 730 (0.25) | 736 (0.25) |  |  |

Q1 is first quartile; Q2 is second quartile; Q3 is third quartile; Q4 is fourth quartile; BMI is body mass index; E-DII is energy-adjusted dietary inflammatory index; HOMA-IR is homeostatic model assessment for insulin resistance; PIR is poverty index ratio; WC is waist circumference; CVD is cardiovascular disease; DM is diabetes mellitus.

**Table** **S2** Risk of depression according to tertiles of DII or E-DII with sensitivity analysis.

|  | Model 1 | Model 2 | Model 3 |
| --- | --- | --- | --- |
|  | *OR* (95% *CI*) | *OR* (95% *CI*) | *OR* (95% *CI*) |
| Tertiles of DII |  |  |  |
| T1 | Reference | | |
| T2 | 1.76 (1.26-2.47) | 1.41 (1.00-1.99) | 1.42 (1.00-2.00) |
| T3 | 2.56 (1.98-3.31) | 1.60 (1.16-2.21) | 1.61 (1.17-2.21) |
| For 1-SD increase | 1.53 (1.37-1.71) | 1.26 (1.10-1.45) | 1.26 (1.10-1.45) |
| *P*-trend | <0.001 | 0.004 | 0.004 |
| Tertiles of E-DII |  |  |  |
| T1 | Reference |  |  |
| T2 | 1.51 (1.14-2.01) | 1.24 (0.94 -1.64) | 1.24 (0.94-1.63) |
| T3 | 2.03 (1.58-2.61) | 1.45 (1.13-1.86) | 1.45(1.13-1.86) |
| For 1-SD increase | 1.42 (1.26-1.61) | 1.23 (1.09-1.40) | 1.23 (1.09 -1.40) |
| *P*-trend | <0.001 | 0.004 | 0.005 |

T1 is first tertile; T2 is second tertile; T3 is third tertile; SD is standard deviation; *OR* is odds ratio; *CI* is confidence interval; DII is dietary inflammatory index; E-DII is energy-adjusted dietary inflammatory index;

Model 1: Did not adjust any covariates;

Model 2: Adjusted for gender, education, age, PIR, CVD, DM, hypertension, BMI, WC, energy intake, alcohol use, serum cotinine.

Model 3: Adjusted for gender, education, age, PIR, CVD, DM, hypertension, BMI, WC, energy intake, alcohol use, serum cotinine, HOMA-IR.

**Table S3** Risk of depression by tertiles of DII or E-DII stratified according to quartile groups of HOMA-IR with sensitivity analysis.

|  | Model 1 | Model 2 |
| --- | --- | --- |
|  | *OR* (95% *CI*) | *OR* (95% *CI*) |
| Tertiles of DII |  |  |
| 0.03 ≤ HOMA-IR< 1.46 |  |  |
| T1 | Reference | |
| T2 | 2.30 (1.18-4.49) | 1.55 (0.82-2.95) |
| T3 | 4.31 (2.59-7.15) | 1.90 (1.04-3.49) |
| 1.46 ≤ HOMA-IR < 2.42 |  |  |
| T1 | Reference | |
| T2 | 1.93 (1.03-3.63) | 1.41 (0.70-2.86) |
| T3 | 3.34 (1.89-5.89) | 1.91 (1.03-3.55) |
| 2.42 ≤ HOMA-IR < 4.20 |  |  |
| T1 | Reference | |
| T2 | 1.07 (0.55-2.06) | 1.03 (0.48-2.18) |
| T3 | 1.73 (0.9 - 3.34) | 1.71 (0.79 -3.68) |
| HOMA-IR ≥ 4.20 |  |  |
| T1 | Reference | |
| T2 | 1.74 (1.09-2.77) | 1.55 (0.94-2.55) |
| T3 | 1.69 (1.09-2.61) | 1.20 (0.62-2.34) |
| Tertiles of E-DII |  |  |
| 0.03 ≤ HOMA-IR< 1.46 |  |  |
| T1 | Reference | |
| T2 | 1.74 (0.96-3.13) | 1.25 (0.67-2.32) |
| T3 | 2.50 (1.44-4.35) | 1.39 (0.77-2.49) |
| 1.46 ≤ HOMA-IR < 2.42 |  |  |
| T1 | Reference |  |
| T2 | 1.47 (0.78-2.80) | 1.40 (0.77-2.54) |
| T3 | 1.88 (0.97-3.65) | 1.60 (0.92-2.79) |
| 2.42 ≤ HOMA-IR < 4.20 |  |  |
| T1 | Reference |  |
| T2 | 1.16 (0.63-2.14) | 0.96 (0.50-1.84) |
| T3 | 2.44 (1.34-4.46) | 1.71 (0.86-3.41) |
| HOMA-IR ≥ 4.20 |  |  |
| T1 | Reference |  |
| T2 | 1.40 (0.87-2.24) | 1.38 (0.84-2.28) |
| T3 | 1.31 (0.83-2.07) | 1.23 (0.76 - 2.02) |

T1 is first tertile; T2 is second tertile; T3 is third tertile; *OR* is odds ratio; *CI* is confidence interval; DII is dietary inflammatory index; E-DII is energy-adjusted dietary inflammatory index; HOMA-IR is homeostatic model assessment for insulin resistance.

Model 1: Did not adjust any covariates;

Model 2: Adjusted for gender, education, age, PIR, CVD, DM, hypertension, BMI, WC, energy intake, alcohol use, serum cotinine.

**Table S4** The details about the quartiles of DII, E-DII, HOMA-IR and the tertiles of DII, E-DII between depressed group and non-depressed group.

| Variable | Depression | |
| --- | --- | --- |
|  | No (*n* = 10,091) | Yes (*n* = 860) |
| Quartiles of HOMA-IR |  |  |
| Q1 | [0.026 ~ 1.443] | [0.173 ~ 1.656] |
| Q2 | (1.443 ~ 2.392] | (1.656 ~ 2.836] |
| Q3 | (2.392 ~ 4.103] | (2.836 ~ 5.388] |
| Q4 | (4.103 ~ 269.413] | (5.388 ~ 128.113] |
| Quartiles of DII |  |  |
| Q1 | [-5.047 ~ -0.202] | [-4.180 ~ 0.574] |
| Q2 | (-0.202 ~ 1.356] | (0.574 ~ 1.973] |
| Q3 | (1.356 ~ 2.635] | (1.973 ~ 3.278] |
| Q4 | (2.635 ~ 5.403] | (3.278 ~ 5.104] |
| Quartiles of E-DII |  |  |
| Q1 | [-5.521 ~ -0.129] | [-4.529 ~ 0.589] |
| Q2 | (-0.129 ~ 1.242] | (0.589 ~ 1.738] |
| Q3 | (1.242 ~ 2.331] | (1.738 ~ 2.795] |
| Q4 | (2.331 ~ 5.259] | (2.795 ~ 5.130] |
| Tertiles of DII |  |  |
| T1 | [-5.047 ~ 0.437] | [-4.180 ~ 0.437] |
| T2 | (0.437 ~ 2.260] | (0.437 ~ 2.255] |
| T3 | (2.260 ~ 5.403] | (2.225 ~ 5.104] |
| Tertiles of E-DII |  |  |
| T1 | [-5.521 ~0.433] | [-4.529 ~ 0.433] |
| T2 | (0.433 ~ 2.000] | (0.433 ~ 2.005] |
| T3 | (2.000 ~ 5.259] | (2.005 ~ 5.130] |

HOMA-IR is homeostatic model assessment for insulin resistance; DII is dietary inflammatory index; E-DII is energy-adjusted dietary inflammatory index; Q1 is first quartile; Q2 is second quartile; Q3 is third quartile; Q4 is fourth quartile; T1 is first tertile; T2 is second tertile; T3 is third tertile.
